# Plasma Levels of Neuron- and Astrocyte-Derived Exosomal Amyloid Beta1-42, Amyloid Beta1-40, and Phosphorylated Tau Levels in Schizophrenia Patients and Non-psychiatric Comparison Subjects: Relationships With Cognitive Functioning and Psychopathology

**DOI:** 10.3389/fpsyt.2020.532624

**Published:** 2021-03-02

**Authors:** Ellen E. Lee, Charisse Winston-Gray, James W. Barlow, Robert A. Rissman, Dilip V. Jeste

**Affiliations:** ^1^Department of Psychiatry, University of California San Diego, San Diego, CA, United States; ^2^Veterans Affairs San Diego Healthcare System, La Jolla, CA, United States; ^3^Department of Neuroscience, University of California San Diego, San Diego, CA, United States; ^4^Department of Psychiatry and Neurosciences, University of California San Diego, San Diego, CA, United States

**Keywords:** serious mental illness, neurodegenerative disease, neurons, astrocytes, cognition, executive functioning

## Abstract

**Introduction:** Cognitive deficits in people with schizophrenia (PWS) are a major predictor of disability and functioning, yet the underlying pathophysiology remains unclear. A possible role of amyloid and tau biomarkers (hallmarks of Alzheimer's disease) is still speculative in schizophrenia. Exosomes or extracellular vesicles, involved with cell-to-cell communication and waste removal, can be used to assay brain-based proteins from peripheral blood. To our knowledge, this is the first study of exosomal amyloid and tau protein levels in PWS.

**Methods:** This cross-sectional study included 60 PWS and 60 age- and sex-comparable non-psychiatric comparison subjects (NCs), age range 26–65 years. Assessments of global cognitive screening, executive functioning, psychopathology, and physical measures were conducted. Exosomes were extracted and precipitated from fasting plasma and identified as neuron-derived exosomes (NDEs) or astrocyte-derived exosomes (ADEs). Human-specific ELISAs were used to assay levels of amyloid-beta 1-42 (Aβ42), amyloid-beta 1-40 (Aβ40), and phosphorylated T181 tau (P-T181-tau). Plasma assays for aging biomarkers (C-reactive protein and F2-isoprostanes) were also performed.

**Results:** ADE-Aβ42 levels were higher in PWS compared to NCs, though the other exosomal markers were similar between the two groups. Higher ADE-P-T181-tau levels were associated with worse executive functioning. Among PWS, higher ADE-P-T181-tau levels were associated with less severe negative symptoms and increased F2-isoprostane levels. Astrocyte-derived Aβ marker levels were sensitive and specific in differentiating between diagnostic groups. Among PWS, Aβ40 levels differed most by exosomal origin.

**Discussion:** Exosomal markers may provide novel insights into brain-based processes (e.g., aging, oxidative stress) from peripheral blood samples.

## Introduction

Cognitive deficits in people with schizophrenia (PWS) are a major predictor of disability and functioning, yet there is limited understanding of the underlying pathophysiology of these impairments. In Alzheimer's disease (AD), it has been hypothesized that pathological amyloid-beta (Aβ) and tau (hallmarks of AD) seed and spread from cell to cell in the brain (1, 2). While the mechanism of the spread is unclear, the Aβ- and tau-based pathology leads to the accumulation of Aβ plaques and neurofibrillary tangles and, ultimately, neurodegenerative changes. Soluble oligomers and intermediate Aβ forms are neurotoxic, and abnormally folded Aβ aggregates form plaques resulting in recruitment and activation of microglial cells, and subsequent inflammation and neurodegeneration. The role of tau has been expanded beyond stabilizing microtubulins to involvement in axonal transport, neuronal signaling, synaptic functioning, as well as DNA transcription and stabilization (3). Tau levels have been linked to aging and neurodegenerative brain changes in older adults (4, 5). Though significant cognitive deficits are common in PWS, the roles of Aβ and tau biomarkers have not been clearly identified in schizophrenia.

In AD, one hypothesized mechanism of the Aβ and tau pathology involves exosomes or extracellular vesicles, involved with cell-to-cell communication, waste removal, and trafficking across the blood-brain barrier. Exosomes can be found in peripheral blood samples, and their specific cellular origin can be identified. The proteins contained in exosomes can be assayed to provide a novel assessment of brain-based proteins using peripheral blood samples. Though the science is its early stages, the preliminary literature in specific disease states including AD and traumatic brain injury shows exosome-based biomarkers to be related to clinical outcomes and to clarify the different roles of cell types (e.g., astrocytes vs. neurons) (6, 7). Furthermore, exosomes provide novel insights into aging processes—including inflammation and oxidative stress (8–10).

Exosome-based Aβ and tau measures have been reported to be representative of cerebrospinal fluid (CSF) and imaging findings among people with AD, people with mild cognitive impairment (MCI), and age-matched controls (11). Unlike peripheral protein levels that depend on the permeability of the blood-brain barrier, exosomal levels may better reflect the intracellular Aβ and tau pathology, specifically within neurons and astrocytes. One study of people with AD found that exosomal Aβ1-42 (Aβ_42_) levels were better correlated with brain plaque deposition on Aβ PET imaging than total circulating levels of Aβ_42_ (12). Furthermore, Neuron-derived exosomal (NDE) Aβ_42_ and P-T181-tau levels were reported to predict conversion from normal cognition or MCI to AD (6, 13). Populations including HIV seropositive individuals have also shown NDE Aβ_42_ levels to be higher in persons with cognitive impairment compared with cognitively normal individuals (14).

Recent studies have supported the potentially important role of exosomes in psychiatric disorders. Exosomal involvement in neuroplasticity (15), trafficking of microRNA (miRNA) (16), and neuroinflammation (17) may have implications for psychiatric disorders. Exosomal biomarkers of neuronal insulin resistance have been shown to be lower in PWS and people with drug-naïve first-episode schizophrenia, and are associated with memory impairments and lower occipital cortex brain glucose utilization (18, 19). One study showed that post-mortem brain exosomal microRNA (miRNA) was differentially expressed in PWS compared to individuals with bipolar disorder and non-psychiatric comparison subjects (16). However, the roles of exosomal amyloid and tau are unclear within PWS, and their associations with cognitive functioning and other biomarkers of aging (i.e., inflammatory and oxidative stress) have not been explored.

Unlike AD, in which studies have generally reported higher plasma Aβ_42_, lower CSF Aβ_42_, and higher plasma and CSF phosphorylated tau levels in individuals with AD compared to cognitively healthy individuals, the literature in schizophrenia is mixed with both similar and differing levels reported in PWS. The majority of post-mortem brain studies found no difference in senile plaques or Aβ_42_ deposition between PWS and non-psychiatric comparison subjects (NCs) (20–25). Consistent with AD, a study of CSF Aβ_42_ reported lower levels in PWS compared to NCs (26, 27). However, studies in PWS, mostly using post-mortem brain data, have not demonstrated a consistent relationship between Aβ_42_ levels and cognitive functioning (28). While CSF levels of tau have not differed between PWS and NCs in three studies (26, 29, 30), one study of tau in peripheral blood found lower total and phosphorylated levels of tau in PWS compared to NCs, which was attributed to the early stage of illness and young age (mean age 39 years) of PWS (31). Two CSF studies of tau levels reported no correlation with cognitive functioning (based on Mini-Mental State Examination scores) (26, 29).

To our knowledge, this is the first study of exosome-derived Aβ and tau protein levels in relationship to cognitive functioning in a sample of younger and middle-aged adults with schizophrenia. We present cross-sectional data from an ongoing study of PWS and NCs. We hypothesized that (1) Plasma exosomal Aβ_42_, Aβ_40_, and P-T181-tau levels will be higher in PWS compared to NCs (similar to AD), and (2) Higher exosomal Aβ_42_, Aβ_40_, and P-T181-tau levels will be correlated with worse cognitive functioning in PWS and NCs. We explored the relationship of exosomal Aβ_42_, Aβ_40_, and P-T181-tau levels with age, sex, psychopathology, physical health measures, as well as peripheral biomarkers of aging: inflammation (high sensitivity C-reactive protein or hs-CRP) and oxidative stress (F2-isoprostanes).

## Materials and Methods

### Study Participants

The study sample has been described in previous papers that reported worse oxidative stress and inflammatory biomarker levels in PWS (32–34). Briefly, this subsample included persons with a diagnosis of schizophrenia or schizoaffective disorder [determined using the Structured Clinical Interview for the DSM-IV-TR (SCID) (35)] and NCs (aged 26–65 years), recruited from the greater San Diego area. NCs were screened with the Mini-International Neuropsychiatric Interview (MINI) (36) for past or present diagnoses of a major neuropsychiatric illness. Other exclusion criteria were: (1) other current DSM-IV-TR Axis I diagnoses; (2) alcohol or other substance abuse or dependence (except tobacco) within 3 prior months; (3) diagnosis of dementia, intellectual disability disorder, or a major neurological disorder; (4) medical disability affecting a subject's ability to complete study procedures. The study protocol was approved by the UC San Diego Human Research Protections Program and all subjects completed consent forms prior to their participation. This subsample included age- and sex-comparable groups of PWS and NC participants with baseline blood samples available for exosomal analyses. There were no other additional selection criteria for the sub-study.

### Clinical Assessments

Trained research staff interviewed and assessed the participants using standardized assessments. Cognitive measures included the Delis-Kaplan Executive Function System for executive functioning (37) and the Telephone Interview for Cognitive Status (modified) (TICS-m) for global cognitive functioning (38). Psychopathology measures included Scales for the Assessment of Positive and Negative Symptoms (SAPS and SANS, respectively) (39, 40). Clinical factors specifically related to schizophrenia included antipsychotic daily dose and duration of illness. Other measures included assessments of smoking (packs per day), alcohol use, mental well-being and physical well-being [Medical Outcomes Survey - Short Form 36 (SF-36)] (41), and medical co-morbidity (Cumulative Illness Rating Scale) (42). Height and waist circumference and weight was measured using standard protocols. Body mass index (BMI) was calculated from the height and weight based on standard formulas.

### Exosomal Assays

The full protocol for isolating and identifying exosomes has been previously described (6, 7). Banked plasma samples were frozen at −80°C in EDTA microcentrifuge tubes.

### Neural Enrichment of L1CAM-Positive Neuronal-Derived Exosomes (NDEs) From Human Plasma via Bead Antibody Exosome (BAE)—FITC Complex and FACS Sort

Two hundred and fifty μL of human plasma were incubated with 2.5 μL purified thrombin (System Biosciences, Inc., Mountain view, CA) at room temperature for 5 min. After centrifugation at 10,000 rmp for 5 min, supernatants were incubated with 63 μL ExoQuick Exosome Precipitation solution (EXOQ; System Biosciences, Inc.), and resultant suspensions were centrifuged at 1,500 x g for 1 h at 4°C. Each pellet was suspended in 300 μl of 1X phosphate buffer saline (PBS) with Halt's protease and phosphatase inhibitor cocktail EDTA-free (Thermo Scientific) followed by immunochemical enrichment of exosomes from neuronal and astrocyte sources.

Neural and astrocyte enrichment was conducted per manufacturer's instructions (System Biosciences, Inc.; Catalog # CSFLOWBASICA-1). Briefly, 40 μL of 9.1 μm, streptavidin magnetic Exo-Flow beads (System Biosciences, Inc.; Catalog # CSFLOWBASICA-1) were incubated with 100 ng/μL of mouse anti-human CD171 (L1CAM, neural adhesion protein) biotinylated antibody (clone 5G3, eBioscience/Thermo Fisher Scientific; Catalog # 13-1719-82) or mouse anti-human GLAST (ACSA-1) biotinylated antibody (Miltenyi Biotec, Inc., Auburn, CA; Catalog # 130-118-984) for 2 h on ice, with gently flicking every 30 min to mix. Bead-antibody (Ab) complex was washed three times in Bead Wash Buffer (Systems Biosciences, Inc.; CSFLOWBASICA-1) using a magnetic stand. Bead-Ab complex was suspended with 400 μL of Bead Wash Buffer and 100 μL of total exosome suspensions rotating overnight at 4°C. Bead-Ab-exosome (BAE) complex was washed three times with Bead Wash Buffer then suspended in 240 μL of Exosome Stain Buffer and 10 μL of Exo-FITC Exosome FACS stain (Systems Biosciences, Inc.; Catalog # CSFLOWBASICA-1) for 2 h on ice, with gently flicking to mix. BAE-FITC complex was washed 3 times in Bead Wash Buffer then suspended in 300 μL of Bead Wash Buffer prior to loading into BD FACS Aria II for sorting.

### Western Blot Protocol

5 μ*g* NDE and ADE preparations were diluted with 4x Laemmli sample buffer (161-0737, BioRad) and resolved on 4–20% Mini-PROTEAN™ TGX™ Precast Protein Gel (456-1096, BioRad) for at 120 mV for 1 h and 15 min. Proteins were transferred to polyvinylidene difluoride (PVDF) membranes using Bio Rad Mini Trans-Blot.

#### Electrophoretic Transfer Cell

Blots were blocked in 5% bovine serum albumin (BSA) in tris-buffered saline (TBS) with 0.1% Tween-20 (TBST) at room temperature for 1 h on a platform shaker. Primary antibodies were applied overnight at 4°C in blocking solution. The primary antibodies used were GFAP Monoclonal Antibody (GA5) (1:000, Catalog # 14-9892-82, eBioscience, Invitrogen, Carlsbad, CA); Anti-NeuN Antibody, clone A60 (1:1000, Catalog # MAB377, Millapore Sigma, Temecula, CA) and Purified Mouse Anti-Flotillin-1 (exosome surface marker; raised against mouse flotillin-1 aa. 312-428, cross reacts with human flotillin-1; 1:100, 610821, BD Transduction Laboratories, San Jose, CA.). Following overnight incubation, blots were washed 3x in 1x TBST then incubated in horseradish peroxidase (HRP) conjugated secondary antibodies (anti-mouse, 1:1000; Thermo-Fisher). Enhanced chemiluminescence (ECL) substrate was applied before visualization by GelDoc (BioRad). Blot analysis was performed on ImageLab 5.2.1 (BioRad).

### Quantification of NDE and ADE Protein Cargo by Human-Specific Enzyme Linked Immunosorbent Assays (ELISAs)

Protein concentrations for eluted NDE and ADE suspensions were determined using the Pierce bicinchoninic acid (BCA) Protein Assay kit (Thermo Fisher Scientific; Catalog # 23225). Mammalian protein extraction reagent (M-PER) (Thermo Fisher Scientific; Catalog # 78501) with protease and phosphatase inhibitors are mixed with eluted NDE and ADE suspensions prior to ELISA quantification. L1CAM-positive NDE cargo proteins and GLAST-positive ADE cargo proteins were quantified by human-specific ELISAs for P-T181-tau (Fujirebio US, Inc., Alpharetta, GA; Catalog # 81582), Aβ_42_ (Meso Scale Discovery (MSD), Rockville, MD; Catalog #K15200E); Aβ_40_ (MSD; Rockville, MD; Catalog # K48500E), and tetraspanning exosome marker CD81 (Cusabio, American Research Products–Waltham, MA; Catalog # CSB-EL004960HU) with verification of the CD81 antigen standard curve using purified human recombinant CD81 antigen (Origene Technologies, Inc., Rockville, MD; Catalog # TP317508), according to suppliers' directions, with the extension of the incubation period to overnight at 4°C, in contrast to 2 h in the instructions. The mean value for all determinations of CD81 in each assay group was set at 1.00, and the relative values for each sample were used to normalize their recovery.

### Blood-Based Inflammatory and Oxidative Stress Biomarkers

As described previously (32), plasma hs-CRP levels were measured with a commercially available (MSD, Rockville, MD) enzyme-linked immunosorbent assay (ELISA) at the Clinical and Translational Research Institute lab (La Jolla, CA).

Also, as described previously (33), the lab assays for F2-isoprostanes levels used gas chromatography/negative ion chemical ionization mass spectrometry (GC/NICI-MS) methodology and normal plasma levels in healthy adults are 0.035 ± 0.006 ng/mL.

### Statistical Analyses

Independent sample *t*-tests, Chi-square tests, and Mann-Whitney U tests were used to assess differences between PWS and NC groups, as well as to explore differences by sex. Extreme outliers on the exosomal biomarkers were identified using GraphPad Prism version 8.0.0 for Windows (GraphPad Software, San Diego, California, USA, www.graphpad.com). The analyses identified 7 outlier values in the NC group and 11 in the PWS group. The individuals who were identified as outliers (on any exosomal measure) in the NC group were comparable by age, education, and all the clinical variables. The outliers from the PWS were older and had more physical comorbidities, but were otherwise comparable by education, smoking, duration of illness, psychopathology, antipsychotic dose, and other clinical measures.

General linear models that controlled for age and sex were used to assess the relationship between cognitive functioning and the biomarkers. Spearman's non-parametric correlations were performed to assess the relationships of exosomal Aβ_42_, Aβ_40_, and P-T181-tau with age and psychopathology measures in each group. Receiver operating characteristic (ROC) analyses were performed to assess the sensitivity of the exosome cargo proteins in differentiating between the NC and PWS groups using GraphPad Prism version 8.0.0 for Windows (GraphPad Software, San Diego, California, USA, www.graphpad.com). ROC analyses were conducted under the non-parametric distribution assumption for standard error of area to determine the performance of classifier models using IBM SPSS Statistics for Windows, version 26 (IBM Corp., Armonk, NY). Assessments of effect size included Cohen's d, correlation coefficients, and ${\text{η}}_{\text{p}}^{2}$.

## Results

**Figure 1:**
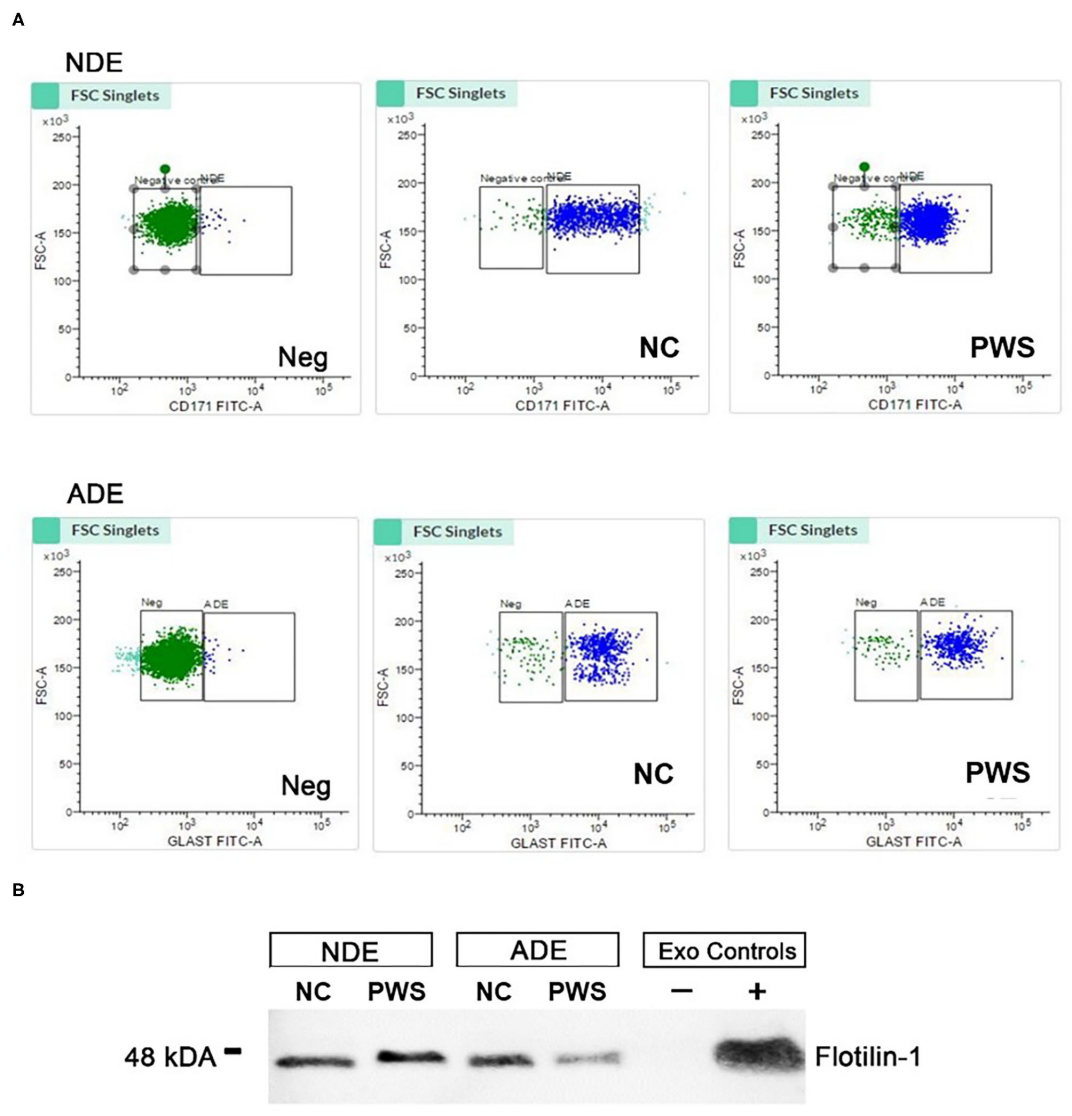
Characterization of neuronal (NDE) and astrocyte derived exosomes (ADE)s. **(A)** FACS analysis of plasma NDEs and ADEs following the formation of bead-antibody-exosome (BAE)—FITC complexes. Streptavidin magnetic beads were incubated with exosomes isolated from non-psychiatric comparison subjects (NC) and people with schizophrenia (PWS) (*n* = 60) and enriched against biotinylated anti-human CD171 biotin (L1CAM, NDE) or anti-GLAST antibody (ADE). BAE complexes are stained with FITC prior to FACS. **(B)** Plasma NDE and ADE preparations from NC and PWS patients were probed with exosome marker, Flotilin-1 (1:1000). Non-exosome fraction (supernant resulting from 1 h spin at 1500G) served as the negative control while total exosomes (diluted in 1× PBS prior to neuronal and astrocyte enrichment) served as the positive control. The resultant Western Blot demonstrated that NDEs are Flotillin and Neun positive and GFAP negative. However, we were not able to get a signal for the ADEs. Due to multiple freeze-thaw cycles, the integrity of the samples used in the current study had diminished significantly.

Plasma exosomes derived from PWS and NC were extracted, precipitated, and enriched against neuronal (L1-CAM) and astrocyte (GLAST/ACSA-1) sources by fluorescent activated cell sorting (FACS) (Figure 1A). Bead-antibody-exosome (BAE)—FITC complexes were generated using respective biotinylated antibodies, which were coupled to magnetic streptavidin beads. Resultant BAE complexes was coupled to a FITC fluorescent tag; that ubiquitously recognizes all exosomes; and subsequently sorted based on green fluorescent intensity (Figure 1A). As a negative control, the resultant non-exosome, bead-antibody-FITC (BA- FITC) complex was sorted to confirm Exo-FITC secondary antibody only binds to exosomes. The significant degree in flow separation from the non-exosome, negative control demonstrates flow cytometry is an efficient method for validating neural and astrocytic enrichment of NDEs and ADEs from human plasma. Additional validation was conducted using Western Blot. Plasma NDEs and ADEs were probed with exosome marker, Flotilin-1 (Figure 1B), neuronal lineage marker, NeuN, and astrocyte marker, Glial fibrillary acidic protein (GFAP) (data not shown). NDE and ADE preparations derived from NC and PWC were positive for Flotilin-1, confirming our preparations are of an exosomal origin however our WB analysis for NeuN and GFAP were inconclusive. We determined that our NDE preparations were positive for NeuN and negative for GFAP however, we were not able to get a positive signal for the ADEs. The non-exosome fraction (resultant supernatant following exosome precipitation) served as the negative control while total exosomes [resultant exosome preparation (diluted in 1x PBS) prior to neuronal and astrocyte enrichment] served as the positive control. In the case of the ADEs, we suspect that sample integrity was significantly compromised due to multiple freeze-thaw cycles thus preventing us from generating an appreciable WB. Moreover, it is plausible that GFAP is not suitable for validating the origin of exosomes derived from astrocytes via WB. GFAP is an intermediate filament protein that may not be present on the surface of ADEs and warrants further review.

**Figure 2:**
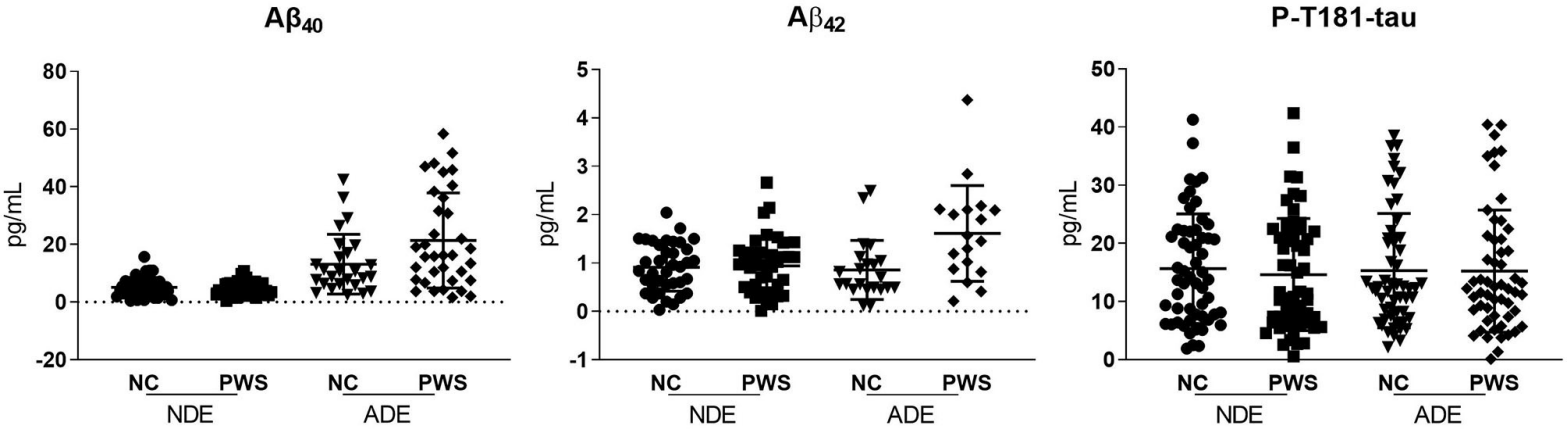
Scatterplots of the exosomal biomarkers. Aβ_40_, amyloid beta1-40; Aβ_42_, amyloid beta1-42; ADE, astrocyte-derived exosomes; NDE, neuron-derived exosomes; P-T181-tau, phosphorylated tau (T181 epitope).

Levels of Aβ_42_ Aβ_40_, and P-T181-tau levels were obtained from ELISA assays. The study sample included 60 PWS (DSM-*IV-TR* criteria) and 60 age- and sex-matched NCs (mean age 48 years, SD 10.4, range 26–65 years) (Table 1). The difference in racial/ethnic backgrounds of the two groups did not meet criteria for statistical significance. PWS had fewer years of education and worse executive functioning and overall cognitive functioning than the NC group. PWS also had worse physical health measures (BMI, physical well-being, comorbidities) and biomarker levels (hs-CRP, IL-6, F2-isoprostanes) than the NCs. PWS group had higher levels of ADE-Aβ_42_ levels, but similar levels of NDE-Aβ_42_ compared with the NC group (Figure 2; Mann Whitney *U*(47) = 387, *p* = 0.02). ADE- and NDE-Aβ_40_, and P-T181-tau levels did not differ between the two groups. There were no significant differences in Aβ_42_ Aβ_40_, and P-T181-tau levels by sex (data not shown).

**Table 1:** Demographics of people with schizophrenia and a non-psychiatric comparison group.

	**Non-psychiatric comparison subjects**			**People with schizophrenia**					
	**N**	**Mean**	**SD**	**N**	**Mean**	**SD**	**Statistical test**	***p***	**Cohen's d**
**Demographic variables**									
Age at visit (years)	60	48.6	10.5	60	48.3	10.3			
Sex (% female)		55			55				
Race/ethnicity							*X*^2^ = 5.71	0.06	
Caucasian (%)		58			37				
Hispanic (%)		23			33				
Other (%)		18			30				
Education (total years)	60	14.3	2.0	60	12.4	2.6	*t*(118) = 4.5	**<0.001**	**0.83**
**Lifestyle factors**									
Smoking (packs per day)	60	0.02	0.08	60	0.39	0.54	*t*(61.3) = −5.4	**<0.001**	–**0.96**
Alcohol use at baseline (%)		20			48		*X*^2^ = 10.7	**0.001**	
**Psychopathology**									
Duration of illness (years)				60	26.6	11.5			
Positive symptoms (SAPS)				59	5.8	5.6			
Negative symptoms (SANS)				59	6.8	6.2			
Antipsychotic dose (mg)				60	1.9	1.6			
**Cognitive functioning**									
Executive functioning (DKEFS)	60	0.5	0.6	59	−0.6	0.8	*t*(117) = 8.0	**<0.001**	**1.47**
Global cognitive screening (TICS-m)	59	38.3	4.6	59	30.9	5.7	*t*(116) = 7.8	**<0.001**	**1.43**
**Physical health**									
Physical well-being (SF-36)	57	52.5	7.4	55	44.0	10.9	*t*(110) = 4.9	**<0.001**	**0.92**
Physical comorbidity	58	2.3	2.6	57	5.8	3.8	*t*(113) = −5.72	**<0.001**	–**1.06**
BMI (kg/m^2^)	55	26.9	5.8	59	31.6	7.0	*t*(112) = −3.85	**<0.001**	–**0.72**
**Blood-based biomarkers**									
hs-CRP (mg/L)	51	1.8	2.0	56	4.9	5.3	*U*(107) = 2128	**<0.001**	
F2 Isoprostanes (ng/mL)	35	0.03	0.02	51	0.04	0.02	*U*(86) = 1203	**0.006**	

*BMI, body mass index; DKEFS, Delis-Kaplan executive function system for executive functioning; hs-CRP, high-sensitivity C-reactive protein; SANS, scales for the assessment of negative symptoms; SAPS, scales for the assessment of positive symptoms; TICS-m, telephone interview for cognitive status (modified). The bolded values are for p < 0.05*.

To examine the relationships of cognitive impairment with amyloid and tau markers, we conducted general linear models of executive functioning and overall cognitive functioning that also included age, sex, and diagnostic group. ADE-P-181-tau levels were associated with executive functioning; such that higher ADE-P-181-tau levels were associated with worse executive functioning in both diagnostic groups (Table 2). Exosomal biomarker levels were not associated with overall cognitive functioning (data not shown).

**Table 2:** Associations of exosomal biomarker levels with executive functioning.

	**B**	**SE**	**F**	**df**	**p**	$\eta_{p}^{2}$
Intercept	0.197	0.332	6.445	1	0.01	0.068
Age (years)	−0.013	0.007	3.296	1	0.07	0.036
Sex (ref: male)	0.257	0.146	3.096	1	0.08	0.034
**Diagnostic group (ref: PWS)**	**0.993**	**0.137**	**52.506**	**1**	**<0.001**	**0.374**
**ADE-P181T-tau**	–**0.014**	**0.007**	**3.886**	**1**	**0.05**	**0.042**
Intercept	0.049	0.360	3.766	1	0.057	0.000
Age (years)	−0.008	0.007	1.243	1	0.269	0.020
Sex (ref: male)	0.220	0.136	2.601	1	0.112	0.040
**Diagnostic group (ref: PWS)**	**1.017**	**0.134**	**57.735**	**1**	**<0.001**	**0.482**
NDE-Aβ_42_	−0.247	0.127	3.795	1	0.056	0.058

*NDE-Aβ_42_, neuron-derived exosomal Aβ1-42; P-T181-tau, phosphorylated tau (T181 epitope); PWS, people with schizophrenia; Statistics, general Linear models of factors associated with executive functioning. The bolded values are for p < 0.05*.

Among the NCs, higher NDE-Aβ_42_ levels were associated with higher BMI (*r* = 0.39, *p* = 0.02) and increased hs-CRP levels (*r* = 0.41, *p* = 0.02). Higher ADE-Aβ_42_ levels were associated with worse physical well-being (*r* = 0.46, *p* = 0.03). Higher ADE-P-T181-tau levels were associated with older age (*r* = 0.35, *p* = 0.009). There were no significant associations between exosomal Aβ or tau with physical comorbidities or biomarker levels.

**Figure 3:**
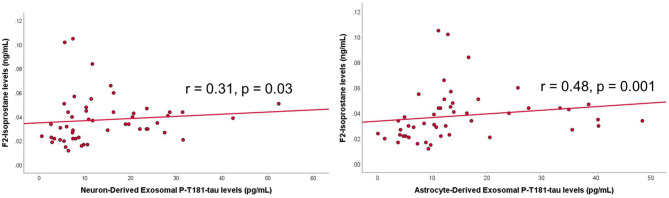
Scatterplots of exosomal tau levels and oxidative stress levels within people with schizophrenia. Spearman's correlation and *p*-value shown on graph. P-T181 tau, phosphorylated tau.

Among the PWS, ADE-P-T181-tau levels were significantly correlated with negative symptoms (*r* = −0.39, *p* = 0.009) such that lower phosphorylated tau levels were associated with worse negative symptoms. ADE- and NDE-P-T181-tau levels were significantly correlated with F2-isoprostane levels, such that higher phosphorylated tau levels were associated with increased oxidative stress (*r* = 0.31, *p* = 0.03 and *r* = 0.48, *p* = 0.001, respectively) (Figure 3). Higher ADE- and NDE-Aβ_40_ levels were associated with worse physical comorbidities (*r* = 0.30, *p* = 0.05 and *r* = 0.35, *p* = 0.05, respectively). Exosomal Aβ and tau measures were not associated with age, BMI, other biomarker levels, antipsychotic daily dose, duration of illness, or positive symptoms.

**Figure 4:**
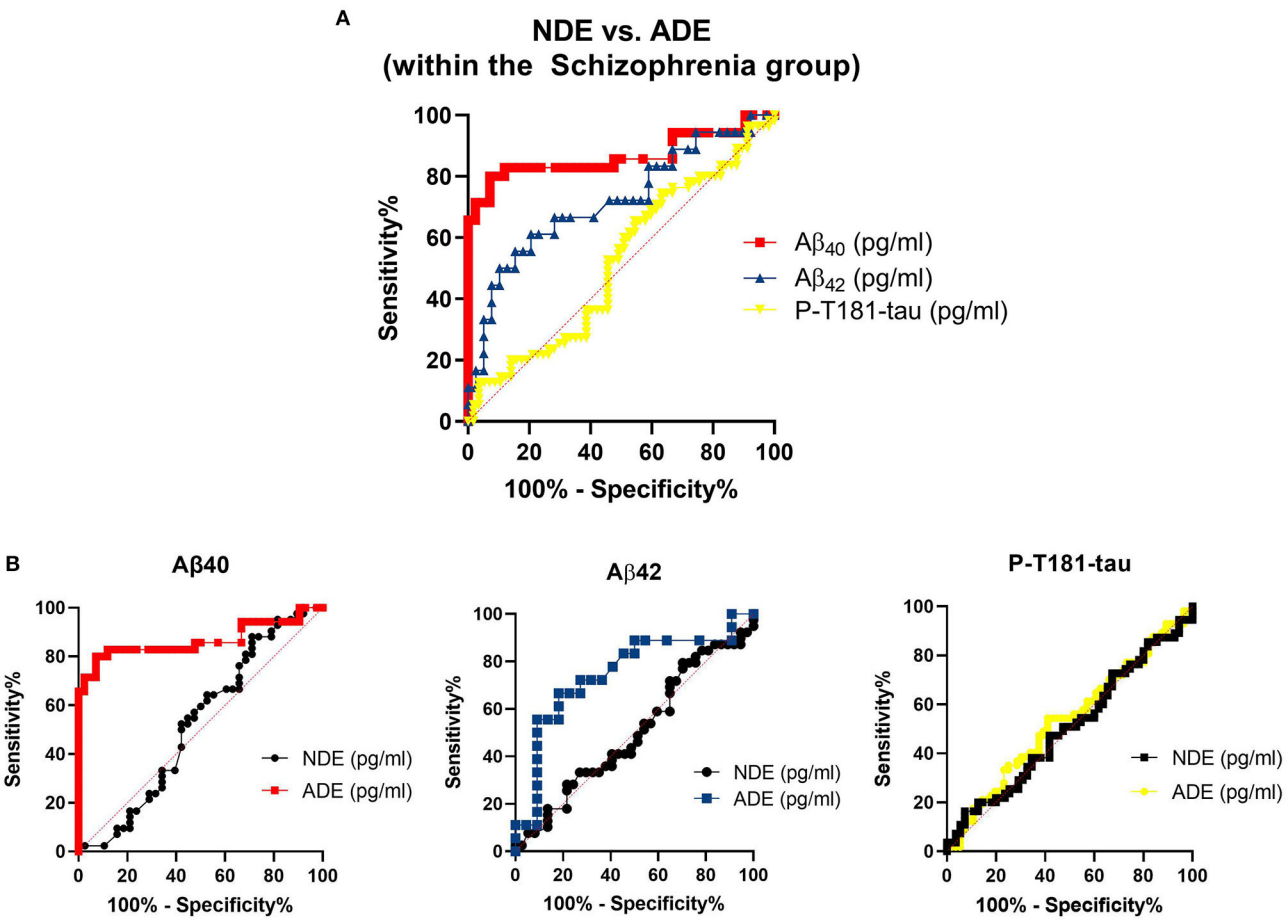
Sensitivity and specificity of exosomal markers to differentiate between diagnostic groups. **(A)** Receiver operating curves (ROCs) and the **(B)** ROC analyses for the exosomal marker sensitivity to distinguish between people with schizophrenia (PWS) and non-psychiatric comparison subjects (NCs). Aβ_42_, amyloid beta1-42; Aβ_40_, amyloid beta1-40; ADE, astrocyte-derived exosomes; NDE, neuron-derived exosomes; P-T181-tau, phosphorylated tau (T181 epitope).

**Figure 5:**
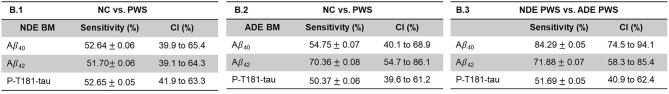
Sensitivity and specificity of exosomal marker levels to differentiate between exosomal origin in people with schizophrenia. Aβ_42_, amyloid beta1-42; Aβ_40_, amyloid beta1-40; ADE, astrocyte-derived exosomes; NDE, neuron-derived exosomes; P-T181-tau, phosphorylated tau (T181 epitope).

The astrocyte-derived exosomal Aβ marker levels appeared to differentiate between the PWS and the NCs better than neuron-derived exosomal Aβ marker levels (Figure 4). While the P-T181-tau levels did not differ as strongly by origin of the exosomes. Among the PWS, levels of Aβ_40_ appeared to differ most strongly by exosomal origin compared to Aβ_40_ and P-T181-tau levels (Figure 5).

## Discussion

Our findings partially supported our hypotheses. Only ADE-Aβ_42_ levels were higher in PWS. In both groups, higher ADE-P-181-tau levels were associated with worse executive functioning. Among the PWS, higher ADE P-T181-tau levels were associated with (surprisingly) lower level of negative symptoms and increased oxidative stress. Tau and Aβ markers were not associated with physical well-being, comorbidities, antipsychotic dose, or duration of illness. Astrocyte-derived exosomal amyloid levels appeared to differentiate between the diagnostic groups, better than the neuron-derived exosomal markers.

Neurons and astrocytes have differing roles in the pathophysiology of schizophrenia. Astrocytes have important roles in producing neurotransmitters, as well as in insulating and maintaining neuronal networks, with impacts on memory and other cognitive functions (43). Astrocyte dysfunction has been linked to schizophrenia pathology, notably the role of astrocytes in producing D-serine and glutamate that could contribute to NMDA receptor hypofunction (44). Studies in persons with traumatic brain injuries (TBI) have also demonstrated contrasting findings between neuronal and astrocyte-derived exosome contents (7).

Interestingly, ADE-Aβ_42_ levels were higher in PWS, which is consistent with exosomal studies in AD and TBI (6, 7) as well as with CSF studies in PWS (26, 27). These findings contrast those of Dwork and colleagues who reported fewer fewer post-mortem senile plaques in PWS (mean age 46 years) who had no documented cognitive impairment based on medical records (45). Cognitive deficits are a common and early symptom of schizophrenia, observed in more than 80% of PWS (46), thus the cohort in the Dwork study may not be representative of many PWS. The lack of difference in tau levels between the groups was consistent with three other studies (26, 29, 30). In contrast to the Demirel et al. study which found lower peripheral tau levels in PWS, the current sample included older PWS (mean age 48 years) with a relatively long duration of illness (mean 26 years) (31). Thus the current findings may reflect the presence of cognitive impairment and older age of the cohort.

Plasma exosomal tau may provide novel insights into cognitive functioning in PWS. The present study found an association between ADE-P-181-tau levels and executive functioning in PWS and NCs, though no relationship was observed with global cognitive screening. While NDE-P-181-tau levels have been shown to be elevated in people with Alzheimer's disease (11, 13), the existing evidence linking ADE-P-181-tau burden with cognition in PWS is limited. Two investigations of post-mortem brains found higher numbers of senile plaques and neurofibrillary tangles in PWS who had moderate-severe cognitive impairment based on medical records, in contrast to PWS without cognitive impairment (25, 45). These studies relied on clinical documentation of cognitive impairment and did not include a standardized measure of cognitive functioning across all the participants. The current study's finding warrant further investigation using assessments of other cognitive domains as well as longitudinal analyses.

While we did not find any previous studies of Aβ levels and BMI in PWS, the AD and aging literature has described conflicting relationships between Aβ levels and BMI. Studies of cortical Aβ on PET imaging consistently reported that higher BMI was associated with lower Aβ burden in older cognitively normal individuals (>60 years), ApoE4 carriers with either mild or no cognitive impairments, and clinically normal older people (age 62–90 years) (47–49). However, the studies of CSF-derived Aβ levels in people with and without cognitive impairment have reported both positive and negative relationships with BMI, with differences based on presence of preclinical Aβ pathology or level of cognitive impairment (50–52). Thus, the links between Aβ and BMI in this study could reflect the impact of preclinical Aβ pathology in the groups or ApoE4 career status and should be interpreted cautiously.

Similarly, the links between exosomal Aβ and hs-CRP levels were only observed in the NCs. Studies of cortical Aβ levels (based on PET imaging) in people with and without AD have reported mixed findings with positive associations with C-reactive protein (CRP) levels in certain subpopulations, e.g., males and Caucasians (53) as well as inverse associations with CRP levels, depending on ApoE4 carrier status (54, 55). Thus, the role of inflammation in relationship to exosomal Aβ and tau may require better characterization of ApoE4 status as well as consideration of other sociodemographic factors.

The links between phosphorylated tau (ptau) and aging have been demonstrated in a number of studies (5). The role of ptau has been expanded from stabilizing microtubulins to involvement in axonal transport, neuronal signaling, synaptic functioning, as well as DNA transcription and stabilization (3). Ptau abnormalities have been linked with neurodegenerative brain changes in older adults (4). The present study only found links between exosomal tau levels and aging in the NCs but not in the PWS. This is similar to a study of CSF tau levels in 19 PWS (age 21–70 years) that reported no relationship with age, though none of the participants had dementia and the subjects were from two discrete age groups (21–38 years and 54–70 years) (29). The association between tau and aging may require further examination, especially in the context of neurodegenerative changes in the general population.

Tau levels were associated with increased oxidative stress and, surprisingly, better negative symptoms in PWS. Increased oxidative stress due to dysregulated metabolism has been observed in early AD, promoting tau phosphorylation and formation of neurofibrillary tangles as well neuronal loss (56, 57). The influence of tau pathology on synaptic connectivity may underlie the neurodevelopmental changes and executive dysfunction associated with schizophrenia (58). Paradoxically, the current findings found higher tau levels to be associated with lower level of negative symptoms. One Turkish study of 42 PWS did not find a relationship between serum tau levels and negative symptoms (31), though there may be discordance between serum and exosomal tau levels. The neurobiology of negative symptoms is unclear due to the variability in symptom persistence and wide heterogeneity of constructs included within negative symptomatology (59). Thus, the exact role of tau pathology in psychotic symptoms warrants further study, with more specific characterization of negative symptoms.

The strengths of this study include age- and sex-comparable groups as well as use of a novel methodology to measure levels of Aβ and tau. At the same time, the study had several limitations. These include the cross-sectional nature of the data. Causality cannot be inferred. Longitudinal examination of the exosomal Aβ relationships to cognition is warranted. Next, this study did not include CSF-derived biomarkers or Aβ PET imaging for correlation. The measure of overall cognitive functioning was a screening tool and does not provide detailed information about specific cognitive domains. Due to multiple freeze-thaw cycles that diminished the integrity of the samples, confirmatory analyses could only be partially completed. Thus, future studies should include analyses on samples that have not been previously thawed. Lastly, this sample included outpatients with schizophrenia on antipsychotics, who had relatively stable, well-controlled psychotic symptoms. Thus, these results may not be generalizable to PWS at two extremes—more acutely ill patients or those who are in sustained remission (60).

While the function of plasma levels of exosomal Aβ and tau and their relationship to CSF protein levels have not been fully elucidated, these markers may be an innovative and relatively non-invasive approach to understanding Aβ and tau in the people with schizophrenia and other psychiatric disorders. Larger and more diverse cohorts will be needed to better examine the relationships between exosomal markers and psychopathology. Exosomal-derived proteins may have future diagnostic and therapeutic implications, and warrant further investigation in the context of psychiatric disorders (61, 62).

## Data Availability Statement

The datasets generated for this study are available on request to the corresponding author.

## Ethics Statement

The studies involving human participants were reviewed and approved by University of California San Diego (UCSD) Human Research Protections Program. The participants provided their written informed consent to participate in this study.

## Author Contributions

EEL, RAR, and DVJ contributed to the conception and design of the study. EEL performed statistical analyses and wrote the first draft of the manuscript. CW-G was involved with data analyses, data interpretation, and writing sections of the manuscript. JWB was involved with the conduct of the exosomal assays and data interpretation. RAR was involved with data analyses and data interpretation. DVJ wrote sections of the manuscript and was involved in data analyses and interpretation. All authors contributed to manuscript revision, read and approved the submitted version.

## Conflict of Interest

The authors declare that the research was conducted in the absence of any commercial or financial relationships that could be construed as a potential conflict of interest.
